# NADPH Oxidase 4 (NOX4) in Cancer: Linking Redox Signals to Oncogenic Metabolic Adaptation

**DOI:** 10.3390/ijms23052702

**Published:** 2022-02-28

**Authors:** Ildiko Szanto

**Affiliations:** Service of Endocrinology, Diabetology, Nutrition and Patient Education, Department of Internal Medicine, Geneva University Hospitals, Diabetes Center of the Faculty of Medicine, University of Geneva, CH-1211 Geneva, Switzerland; ildiko.szanto@unige.ch; Tel.: +41-22-379-5238

**Keywords:** reactive oxygen species, NADPH oxidase, metabolism, cancer, oncogenesis

## Abstract

Cancer cells can survive and maintain their high proliferation rate in spite of their hypoxic environment by deploying a variety of adaptative mechanisms, one of them being the reorientation of cellular metabolism. A key aspect of this metabolic rewiring is the promotion of the synthesis of antioxidant molecules in order to counter-balance the hypoxia-related elevation of reactive oxygen species (ROS) production and thus combat the onset of cellular oxidative stress. However, opposite to their negative role in the inception of oxidative stress, ROS are also key modulatory components of physiological cellular metabolism. One of the major physiological cellular ROS sources is the NADPH oxidase enzymes (NOX-es). Indeed, NOX-es produce ROS in a tightly regulated manner and control a variety of cellular processes. By contrast, pathologically elevated and unbridled NOX-derived ROS production is linked to diverse cancerogenic processes. In this respect, NOX4, one of the members of the NOX family enzymes, is of particular interest. In fact, NOX4 is closely linked to hypoxia-related signaling and is a regulator of diverse metabolic processes. Furthermore, NOX4 expression and function are altered in a variety of malignancies. The aim of this review is to provide a synopsis of our current knowledge concerning NOX4-related processes in the oncogenic metabolic adaptation of cancer cells.

## 1. Introduction

**Figure 1:**
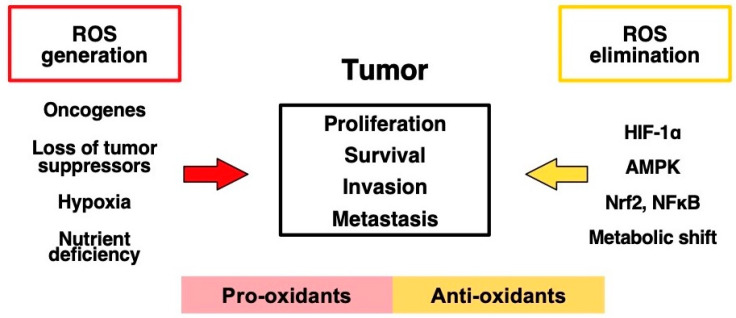
Cellular redox homeostasis in cancerous cells. Cancer cells display enhanced ROS production due to oncogene activation or loss of tumor suppressors and a relative oxygen and nutrient deficiency. To combat the onset of oxidative stress, cancer cells employ different defense mechanisms by activating transcription factors (HIF1α, Nrf2, NFκΒ) to enhance the transcription of pro-angiogenic and antioxidant genes. In addition, these factors also modulate metabolic gene expression to favor a metabolic shift to support the increased need for biomolecules and antioxidant molecules. AMPK is a sensor of nutrient status and can also support metabolic reorientation. Taken together, these pro- and antioxidant components promote tumor proliferation, survival, invasion, and metastasis [3]. ROS: reactive oxygen species; HIF1α: hypoxia inducible factor 1 alpha; AMPK: AMP-activated protein kinase; Nfr2: Kelch-like ECH-associated protein 1 (Keap1)-Nuclear factor erythroid 2-related factor 2; NFκΒ: nuclear factor kappa-light-chain-enhancer of activated B cells.

Cancer initiation, progression, and propagation are multifactorial and intertwined events. One of the components of this intricate network is the cellular redox balance that is sustained by an equilibrium between the production and elimination of reactive oxygen species (ROS) [1,2,3]. ROS play an essential role in the modulation and maintenance of oncogenic transformation at different levels. Indeed, ROS modulate the rewiring of cellular metabolic pathways that are critical for cancer cell survival and proliferation [4,5,6]. In addition, redox cues modify the composition of cancerous cells’ extracellular environment, the ingrowth of capillaries, and immune cell responses; thus, they contribute to the modulation of the tumor’s metastatic capacity and therapy resistance [7,8,9] (Figure 1).

One of the cellular ROS sources enticing emergent interest in these oncogenic processes is the family of NADPH oxidase enzymes (NOX-es) [10,11,12]. NOX-es produce ROS as their unique enzymatic activity and are present in diverse organs with a cell-specific expression pattern. NOX-es deliver ROS in response to specific extra- and intracellular signals in a timely and spatially controlled manner and thus regulate a plethora of physiological processes [13,14]. However, a disruption of this coordinated NOX-derived ROS production is associated with multitudinous pathological alterations, among them cancerogenesis [10,11,12,15]. In this regard, one of the most studied members of the NOX family is the isoform NOX4. NOX4 is characterized by ubiquitous expression and continuous hydrogen peroxide (H_2_O_2_) production, implying a generalized role for NOX4 in the maintenance of basal physiological redox homeostasis. The activity of NOX4 can be enhanced by hypoxia as well as by instigating its mRNA transcription and/or protein translation [16,17,18,19]. Elevated NOX4 mRNA and protein levels have been identified in cancers of diverse origins [10,20,21,22,23,24,25,26,27]. 

Two major coordinators of oncogenic metabolic adaptation are the hypoxia inducible factor 1 (HIF-1) and AMP-activated protein kinase (AMPK), and in certain tumor types (e.g., glioblastoma, renal and gastric cell carcinomas) NOX4 has been recognized as a modulator of their signaling [28,29,30,31,32,33,34]. NOX4 is also entailed in the activation of the redox-sensitive transcription factors NFκΒ and Nrf2 (nuclear factor kappa-light-chain-enhancer of activated B cells and Kelch-like ECH-associated protein 1 (KEAP-1)-Nuclear factor erythroid 2-related factor 2, respectively) [35,36]. Both NFκΒ- and Nrf2-mediated gene transcriptions are integral parts of cancer cell antioxidative defense mechanisms and metabolic reorientation [37,38]. Redox pathways and NOX enzymes in particular are increasingly recognized as important features of cellular oncogenic reprogramming that provide therapeutic possibilities to combat cancer proliferation and invasion [39,40,41,42,43]. This review assembles recent data on redox signaling in the adaptation of cancer cell metabolism with a specific focus on the role of NOX4 in these processes. 

## 2. Regulation of Cellular Redox Homeostasis

### 2.1. Reactive Oxygen Species and Cellular Antioxidant Systems

ROS are short-lived, chemically highly reactive, oxygen-containing molecules, encompassing both oxygen radicals and non-radical molecules [44]. ROS are produced as by-products in a continuous fashion by the mitochondria, the peroxisomes, microsomal P450 enzymes, xanthine oxidase, cyclooxygenases, and lipoxygenases [45]. ROS can also be generated in a purposeful manner by NOX enzymes that provide ROS in response to specific physiological cues in a timely and spatially defined way [46,47,48]. The biologically most significant cellular ROS are superoxide (O_2_^−^), hydrogen peroxide (H_2_O_2_), and the hydroxyl radical (OH) [44]. Superoxide is generated by the loss of an oxygen molecule and is rapidly converted into H_2_O_2_ spontaneously or in an enzymatical reaction catalyzed by superoxide dismutase (SOD). Hydrogen peroxide can also be directly produced by peroxisomes, NOX4, and the Dual Oxidases 1 and 2 (DUOX1 and DUOX2) [49,50,51]. Superoxide can interact with H_2_O_2_ in metal ion-assisted reaction, resulting in the formation of the highly toxic OH [52]. The different ROS types are characterized by different strengths of chemical reactivity, half-life, and diffusion capacity. Based upon these features, H_2_O_2_ is considered as the most relevant cellular signaling ROS due to its relatively long life (10^−5^ s) when compared to other ROS species, a wide range of chemical reactivity (10^−8^ μM: proliferation inducer, 10^−6^ μM: growth arrest inducer, and 10^−4^ μM: apoptotic effect), and the capacity to traverse biological membranes due to its neutral charge at physiological pH values [53,54]. Hydrogen peroxide can also be transported by Aquaporin channels through lipid membranes [54].

**Figure 2:**
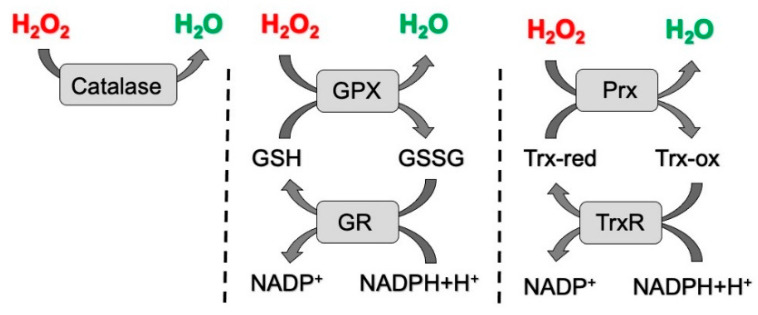
Cellular H_2_O_2_ elimination systems. Cellular H_2_O_2_ elimination can occur through different enzyme systems that allow the conversion of H_2_O_2_ into H_2_O [56]. GPX: glutathione peroxidase; GSH: reduced form of glutathione; GSSG: glutathione disulfide, oxidized form of glutathione; GR: glutathione reductase; Prx: peroxiredoxin proteins; Trx-red: reduced form of thioredoxin; Trx-ox: oxidized form of thioredoxin; TrxR: thioredoxin reductase.

Cellular ROS originating from diverse cellular sources are promptly removed to either prevent redox damage to biomolecules and cellular organelles or to terminate signaling events [55,56]. ROS removal occurs through a network of antioxidant systems that comprise of SOD, catalase, and the glutathione, thioredoxin (Trx), and peroxiredoxin (Prx) systems [57]. SOD enzymes convert superoxide anions to H_2_O_2_ and molecular oxygen in the cytosol (SOD1), the mitochondria (SOD2), and in the extracellular space (SOD3) [58,59,60]. Catalase mediates the reduction of H_2_O_2_ to H_2_O and oxygen. Catalase plays a major role in the degradation of H_2_O_2_ produced during the peroxisomal catabolism of very long-chain fatty acids and also eliminates H_2_O_2_ derived from NADPH oxidases that is produced at plasma membranes [49,61,62]. In addition to catalase, the elimination H_2_O_2_ relies mainly on the glutathione and peroxiredoxin/thioredoxin systems that degrade H_2_O_2_ in a coordinated and cyclic manner through a series of oxidation/reduction reactions [63,64,65]. First, H_2_O_2_ is reduced by glutathione peroxidase (GPX) by oxidizing the free sulfhydryl (-SH) groups of glutathione (GSH) and converting it into glutathione disulfide (GSSG). Then, GSH is regenerated for the next cycle of utilization by glutathione reductase (GR) employing NADPH as an electron donor. In addition to glutathione, H_2_O_2_ can also be degraded by the peroxiredoxin/thioredoxin system. Peroxiredoxins represent a family of six small thiol proteins that catalyze the reduction of H_2_O_2_ to H_2_O both in the mitochondria and in the cytoplasm while they undergo oxidation [66]. Oxidized Prx proteins are subsequently regenerated to their reduced form by thioredoxins. Thioredoxins (Trx) are disulfide-containing proteins that are maintained in a reduced state by thioredoxin reductase (TrxR) in a NADPH-dependent reaction [67]. The components of the cellular H_2_O_2_ antioxidant defense systems are depicted in Figure 2. 

Maintenance of a proper equilibrium between the antagonistic actions of ROS producing and eliminating systems is primordial for healthy cellular functions [68]. An inequity between the capacities of the cellular pro- and antioxidant systems will lead to disturbed redox homeostasis and promote the onset of oxidative or reductive stress [69,70,71,72,73]. Both stress conditions adversely affect cellular activities that are vital for the proper control of cell stemness, proliferation, and metabolic adaptation, as well as the regulation of interaction between the cell and its environment [1,74,75,76,77,78,79]. The clinical relevance of oxidative/reductive stress in oncogenic processes is well documented and is considered to be a compelling therapeutic target in diverse cancer types [1,76,80]. One of the cellular ROS sources whose perturbed functions have been related to oncogenesis is the family of NOX enzymes.

### 2.2. The Family of NADPH Oxidase Enzymes 

ROS, when generated in a timely and spatially controlled fashion, are key elements in the physiological regulation of various cellular functions and receptor signaling events [81,82,83,84,85]. One of the major sources of these cellular signaling ROS are the members of the NOX/DUOX family enzymes that are homologues of the phagocyte NADPH oxidase gp91^phox^/NOX2. There are five NOX and two DUOX isoforms termed NOX1, NOX2, NOX3, NOX4, and NOX5, and DUOX1 and DUOX2, respectively. NOX/DUOX enzymes are expressed in a cell-specific manner and beside the mitochondria constitute one of the major sources of intracellular ROS [10,50,86]. Structurally, all NOX enzymes are six-transmembrane proteins with a conserved core element containing four heme-binding histidines that allow trans-membrane electron transport and two cytoplasmic C-terminal sites that bind NADPH and FAD. Regulation of activity of NOX/DUOX enzymes is achieved through different molecular mechanisms allowing for controlled ROS production in response to specific physiological cues [13,50,87,88]. 

### 2.3. The NADPH Oxidase 4 (NOX4)

**Figure 3:**
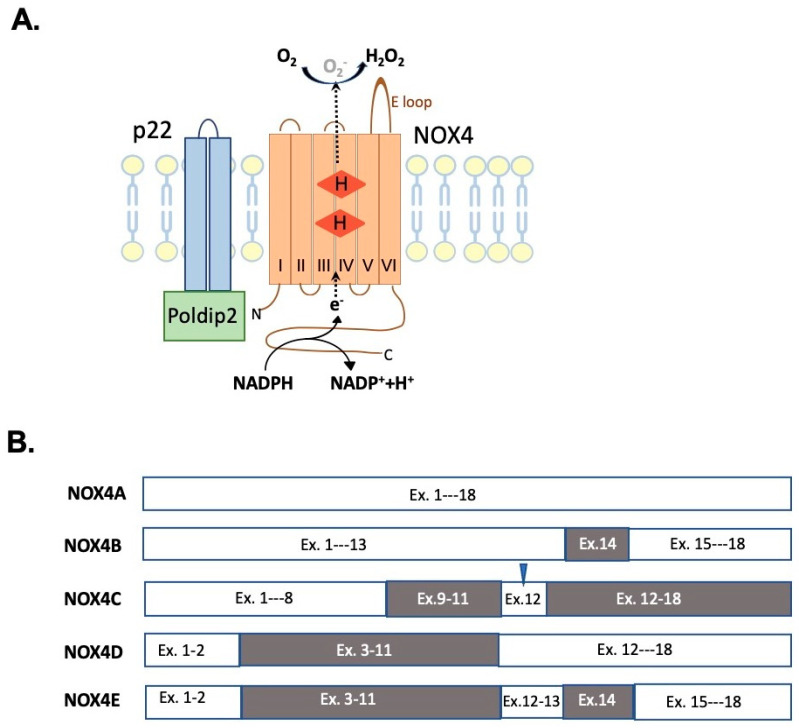
(**A**) Structure of NOX4. NOX4 is comprised from six membrane-spanning alpha-helices (I–VI) connected by five loops (A–E) and an intracytoplasmic tail that contains binding sites for FAD (not shown) and NADPH. Two heme molecules (H in red box) are anchored to four histidine residues in helices III and V, allowing electron transport to oxygen molecules. H_2_O_2_ production takes place in the E loop. The protein p22 supports the stabilization of NOX4, while Poldip2 associates with p22 and contributes to the activation of NOX4 [92,93]. (**B**) NOX4 transcript variants [94]. Ex. = exon, the numbers refer to the numeration of exons. Empty box: exons present in the mRNA variant. Shaded boxes: missing exons from the mRNA variant. Triangle: early STOP codon.

Historically, NOX4 was cloned from the kidney and thus, in initial publications, it was referred to as “Renox” [89,90]. However, subsequent studies established the presence of NOX4 in a wide variety of tissues and cell types, and currently, NOX4 is considered as a ubiquitously expressed NOX isoform [91]. NOX4 forms a heterodimer with p22^phox^ that is necessary for NOX4 expression and activation [92]. The NOX4/p22^phox^ complex also associates with Poldip2 (polymerase [DNA-directed] delta-interacting protein 2) that acts as a potent positive regulator of NOX4 activity [93]. The structure of NOX4 is depicted in Figure 3A. The full-length NOX4 mRNA codes for a protein of 67kDa that shares only 39% amino acid identity with the prototype phagocyte NOX2 [87]. The human NOX4 mRNA gives rise to five different splice variants with different ROS-producing capacities when analyzed in a heterologous overexpressing cellular system in vitro [94] (Figure 3B). However, currently, little knowledge is available concerning the in vivo cellular and intracellular expression pattern of these isoforms and their physio-pathological relevance in different NOX4-mediated effects. The splice variants NOX4B and C lack the NADPH binding site and both the NADPH and FADH binding sites, respectively. Consistently, NOX4B and C act as dominant negative isoforms [94]. The other two NOX4 isoforms, NOX4D and E, are devoid of transmembrane domains, implying that these variants are not membrane-associated. NOX4E is also deficient in the NADPH binding domain and thus, it is uncapable of ROS generation. On the contrary, NOX4D possesses ROS producing capacity that is comparable to the full-length NOX4 (NOX4A) [94]. Anilkumar et al. identified NOX4D as a nucleus resident isoform that mediates the redox-related upregulation of mitogen-activated kinase (MAPK) activity in vascular cells [95]. Importantly, the presence of NOX4D protein in nuclear membranes was demonstrated in selected acute myeloid lymphomas, and NOX4D-mediated H_2_O_2_ production was an essential contributor to the genetic instability and aggressive phenotype of these tumors [96].

NOX4 possesses a constitutive activity, which is principally regulated at the transcriptional/translational levels [17,18]. In addition, however, recent data indicated that NOX4-derived ROS production can be induced by hypoxia [16]. Interestingly, NOX4 releases H_2_O_2_, but the exact mechanism of how NOX4 directly converts superoxide into H_2_O_2_ without a bona fide peroxidase domain remains at present incompletely understood [97]. The physiological importance of immediate H_2_O_2_ release is supported by the fact that NOX4-mediated activation of MAPK was absent in superoxide-generating NOX4 mutants [97]. The continuous ROS-producing activity of NOX4 implies an important role for NOX4 in the regulation of basal cellular redox tone [89,90,98]. Within the cell, the local production and concentration of H_2_O_2_ are critical in determining its effects [99,100]. The intra-cellular localization of NOX4-derived H_2_O_2_ production appears to be cell-type dependent. For example, NOX4 protein has been detected in the endoplasmic reticulum (ER) and nuclei of human airway smooth muscle cells and vascular endothelial cells [25,101], in focal adhesions in vascular smooth muscle cells [102], in the mitochondria of renal mesangial and endothelial cells [103], and in association with the cellular actin network [104,105]. These data are in line with the implication of NOX4 in the regulation of ER stress, DNA damage, the modification of EC matrix, and mitochondrial ROS production as well as cell tonicity and motility. Interestingly, the relationship between mitochondria and NOX4 are bidirectional. Indeed, mitochondrial ATP produced through OXPHOS limits NOX4 activity by binding to a specific ATP-binding motif in the C-terminus tail of NOX4 [106]. Conversely, NOX4 represses mitochondrial biogenesis and Complex I activity [107,108].

## 3. NOX4 in Tumor Cell Hypoxia, Redox Milieu and Metabolic Adaptation

**Figure 4:**
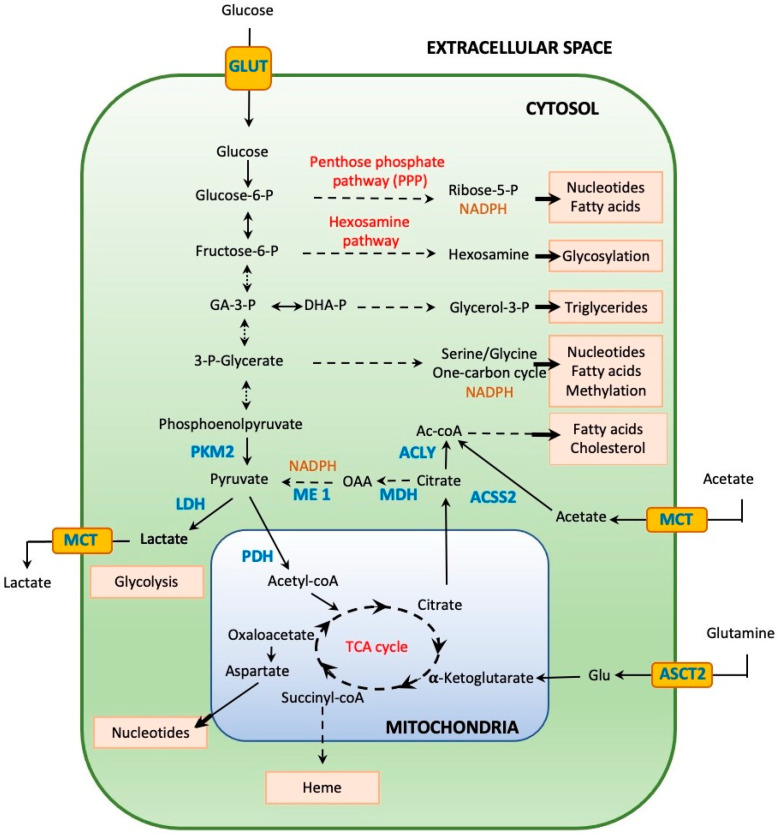
Major elements of cancer cell metabolism rewiring. Cancer cells rewire their metabolic fluxes in order to accommodate their augmented need of biomass synthesis in order to support their high proliferation rate in a hypoxic environment. Glucose is reoriented toward glycolysis, producing pyruvate, which can be reduced to lactate and secreted in the extracellular space through the MTC1 transporter or be carried into the mitochondria to be converted into acetyl-coA to feed the TCA cycle. The rate-limiting activity of PKM2 leads to the accumulation of diverse glycolytic intermediaries fueling the pentose phosphate and hexosamine pathways to support nucleotide and fatty acid synthesis and glycosylation processes, respectively. Other intermediaries (GA-3-P and 3-P-glycerate) are channeled toward the synthesis of triglycerides, nucleotides, or fatty acids, or the production of molecules implicated in DNA or protein methylation reactions. Intermediate molecules of the TCA cycle (aspartate, succinyl-coA) can serve as precursors for nucleotides or heme. Mitochondrial citrate can be shuttled into the cytoplasm and converted into pyruvate to boost NADPH production or deconstructed to acetyl-coA to fuel fatty acid and cholesterol fabrication. Cancer cells preferentially use glutamine and acetate as alternative energy sources that are transported through specific membrane transporters (MCT and ASCT2) and are incorporated into the TCA cycle or acetyl-coA, respectively (reviewed in [119]). Several pathways are implicated in NADPH production (see Figure 5 for details). Dashed arrows indicate multiple step processes; solid arrows indicate direct one-step reactions; critical enzymes are marked in blue. Glycolytic intermediaries: Glucose-6-P: glucose-6-phosphate, Fructose-6-P: fructose-6-phosphate, GA-3-P: glyceraldehyde-3-phosphate, DHA-P: dihydroxyacetone phosphate, glycerol-3-P: glycerol-3-phosphate. Transporters: GLUT: glucose transporter, MTC: monocarboxylate transporter, ASCT2: solute carrier family 1 member 2. Enzymes: PDH: pyruvate dehydrogenase, MDH: malate dehydrogenase, ACLY: ATP citrate lyase, ME1: malic enzyme 1, LDH: lactate dehydrogenase, PKM2: pyruvate kinase M2, ACSS2: acyl-coenzyme A synthetase short-chain family member 2. TCA cycle: Krebs tricarboxylic acid cycle.

Hypoxia, a reduction in cellular oxygen levels due to inadequate oxygen supply by insufficient or malformed capillaries, is one of the hallmarks of solid tumors [109]. Tumor hypoxia bears significant clinical importance, as it is associated with more aggressive tumor growth and poor therapeutic outcomes [110,111,112]. In physiological conditions, hypoxia entices a shift in cellular metabolism that ensures cell survival and a protection against oxidative stress. However, this adaptive mechanism is also exploited by cancerous cells, allowing them to thrive in conditions with insufficient oxygen and nutrient supplies compared to the needs of their unbridled cellular growth [113,114]. In addition, these processes enable cancer cells to evade immune surveillance and to modify their extracellular environment to support invasion and metastasis [115,116]. One of the major hypoxia-related adaptive metabolic mechanisms is the derivation of cellular glucose metabolism toward anaerobic pathways (glycolysis and lactate production) while lessening the oxygen-reliant mitochondrial ATP production [117,118]. Channeling pyruvate toward lactate has another advantage, as it prevents the accumulation of cytosolic NADH and reduces ATP production, thus promoting continuous glucose utilization by limiting the negative feedback effects of NADH and ATP. In addition, this metabolic reorientation also promotes the production of reducing equivalents (NADPH and reduced glutathione); therefore, it boosts the cellular antioxidant capacity and affords biomolecule synthesis for proliferation [75,119,120] (Figure 4).

NADPH functions as a key reducing factor in several biosynthetic pathways as well as in the reactions of two major antioxidant systems: the glutathione and Trx/Prx antioxidant complexes [121]. NADPH is membrane-impermeable and is produced in a compartmentalized fashion in the cytosol as well as in the mitochondria to supply for local need [122,123,124,125]. In the cytosol, the majority of NADPH is derived from the pentose phosphate pathway (PPP) through the reduction of NADP^+^ in two sequential reactions catalyzed by glucose-6-phosphate dehydrogenase (G6PDH) and 6-phosphogluconate dehydrogenase (PGD). Other cytosolic processes that generate NADPH are the reactions catalyzed by malic enzyme 1 (ME1) and isocitrate dehydrogenase 1 (IDH1) [126]. In the mitochondria, NADPH can be produced by mitochondrial isoforms of ME and IDH (ME3 and IDH2, respectively). Of particular interest for cancer metabolism is the contribution of mitochondrial serine/glycine/folate metabolism to NADPH formation and antioxidant defense through glutathione (a tripeptide consisting of cysteine, glycine, and glutamic acid) synthesis [126]. Indeed, cancerous cells divert approximately 10% of the 3-phosphoglycerate generated in glycolysis toward 3-phosphohydroxy-pyruvate, a precursor of serine biosynthesis. Then, serine is converted to glycine and used for refueling the one-carbon metabolism that provides a variety of essential components for macromolecule synthesis. Concerning redox homeostasis, the complex cycles of the one-carbon metabolism provide cytosolic NADPH and glutathione (reviewed in detail in [127]). In addition, to its role in the one-carbon metabolism, serine is also an allosteric activator of the pyruvate kinase 2 (PKM2), an isoform of pyruvate kinase (PK) predominantly expressed in proliferating tissues. The role of PKM2 in cancerogenesis is complex, but one of the most relevant aspects is that PKM2 shifts glucose catabolism away from the normal mitochondrial respiratory chain toward lactate production, thus contributing to tumor cell metabolic adaptation and supporting their proliferation capacity [128]. 

**Figure 5:**
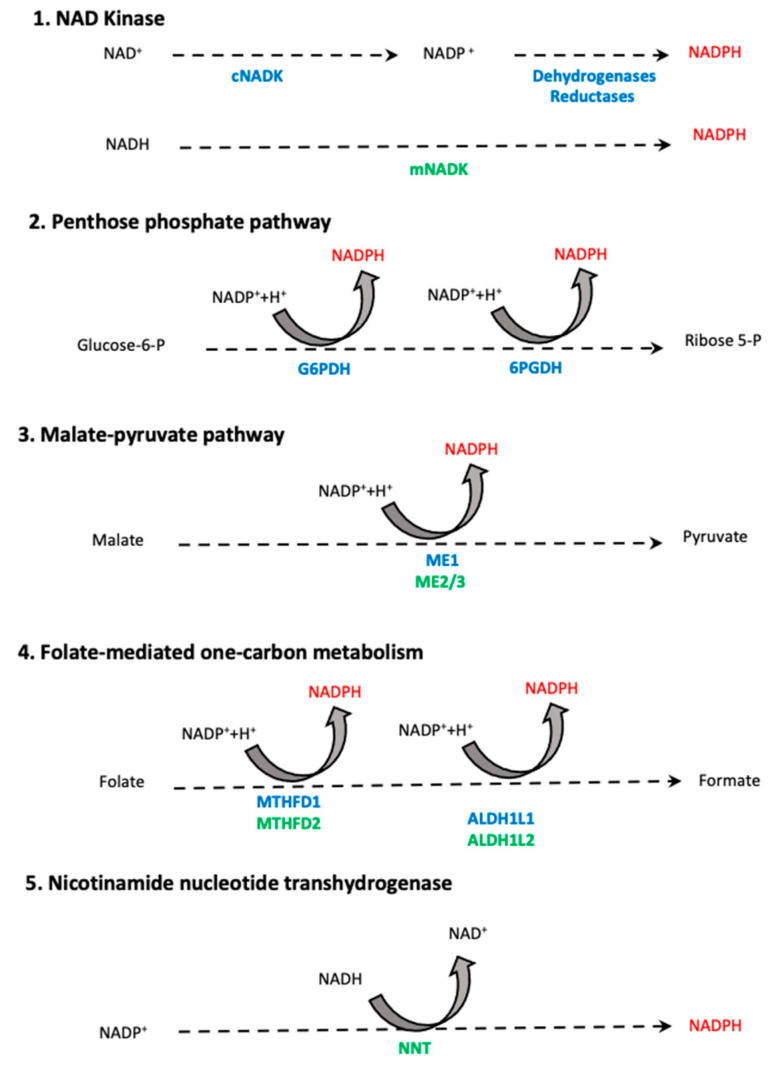
Schematic representation of cellular NADPH-producing pathways. NADPH is mainly produced in glycolytic processes through the pentose phosphate pathway and during malate–pyruvate conversion. In addition, the complex cytosolic and mitochondrial folate-mediated one-carbon cycles provide NADPH. Lesser amounts of NADPH are provided by the direct phosphorylation of NAD^+^ and NADH. In the mitochondria, the enzymatically catalyzed reduction of NADP^+^ by NADH can provide local NADPH (reviewed in [5]). Dotted lines indicate multistep processes, cytosolic enzymes are marked in blue, mitochondrial enzymes are marked in green. cNADK: cytosolic nicotinamide adenine dinucleotide phosphate kinase, mNADK: mitochondrial nicotinamide adenine dinucleotide phosphate kinase, G6PDH: glucose-6-phosphate dehydrogenase, 6PGDH: 6-phosphogluconate dehydrogenase, ME1: malic enzyme 1, ME2/3: malic enzyme 2/3, ALDH1L1: 10-formyltetrahydrofolate dehydrogenase 1, ALDH1L2: 10-formyltetrahydrofolate dehydrogenase 2, MTHFD1: methylenetetrahydrofolate dehydrogenase 1, MTHFD2: methylenetetrahydrofolate dehydrogenase 2, NNT: nicotinamide nucleotide transhydrogenase.

Cellular NADPH production is compartmentalized; however, mitochondrial NADPH synthesis still can contribute to the maintenance of cytosolic NADPH pool through substrate transport, e.g., citrate that can ultimately be converted into α-ketoglutarate with concomitant NADPH release [129]. The diverse cytosolic and mitochondrial NADPH producing pathways are depicted in Figure 5. Taken together, this metabolic rewiring allows the efficient utilization of available nutrients and provides sustained production of the components of the antioxidant systems to prevent the accumulation of harmful oxidants. The importance of these metabolic changes in oncogenesis is underlined by data showing that enzymes catalyzing these reactions are often overexpressed or underwent gain-of-function mutations in cancer cells [120]. In addition, the relevance of these enzymes as pharmacological targets is reflected in the numerous clinical trials in oncological settings [130]. 

Hypoxia-induced cellular responses are essentially coordinated by the hypoxia-inducible transcription factors (HIFs) and the AMP-activated protein kinase (AMPK) [131,132]. Enhanced signaling by HIFs and AMPK has been identified in diverse tumors and linked to the cancerous rewiring of cellular metabolic processes [133,134].

HIFs function as primary oxygen-sensing transcription factors comprising of an oxygen-sensitive α subunit (HIF-1α or HIF-2α) and a constitutively expressed β subunit (HIF-β), also termed as aryl hydrocarbon receptor nuclear translocator (ARNT) [135]. Under normoxic conditions, the alpha subunit undergoes sequential hydroxylation and polyubiquitination, eliciting its degradation via the 26S proteasome. Hypoxia prevents HIF-1α hydroxylation, allowing the stabilized molecule to translocate into the nucleus. Once in the nucleus, HIF-α dimerizes with the HIF-1β/ARNT subunit and additional co-activators initiating its binding to hypoxia-responsible elements (HREs) and prompting the transcription of target genes [133]. The most notable of these target genes is the vascular endothelial growth factor (VEGF) that is mandatory for neo-capillary formation and diverse metabolic enzymes [117,118]. The relationship between ROS and HIF-1 is bidirectional. Indeed, ROS can stabilize HIF-1α and thus increase its transcriptional activity by interfering with its hydroxylation and ubiquitination [135]. On the contrary, HIF-1α can induce transcriptions of ROS eliminating genes (SOD and catalase) and divert metabolic pathways to replenish the levels of reduced glutathione leading to enhanced ROS eradication [118]. The crucial role of HIF-1α-mediated metabolic switch in the defense against redox stress is supported by in vitro data demonstrating that embryonic fibroblasts derived from HIF-1α-deficient mice fail to convert from oxidative to glycolytic metabolism when placed in hypoxic conditions and ultimately die due to excessive ROS accumulation [136]. HIF protein levels are increased in diverse cancer types [137]. The clinical relevance of elevated tumor HIFs levels are demonstrated by their association with poor outcomes in gastric cancer, hepatocellular carcinoma, and breast cancer [138,139,140]. HIF-1 inhibitors are considered as viable agents in the treatment of advanced or refractory cancers, and several compounds are currently undergoing clinical trials [141].

NOX4 is a target gene of the hypoxia-sensitive transcription factor HIF-1α [142]. Conversely, NOX4-derived H_2_O_2_ is necessary for the hypoxia-related stabilization of both HIF-1α and HIF-2α [32,92,143,144,145]. HIF-1-mediated mRNA upregulation of glucose transporter 1 (GLUT1), lactate dehydrogenase A (LDHA), and pyruvate kinase isoform M2 (PKM2) required NOX4-derived ROS production to promote glycolytic switch in thyroid and in diverse non-small cell lung cancer cell lines [92,146]. In addition, in thyroid cells, NOX4 acted as a glycolytic regulator through mitochondrial ROS production to sustain thyroid cancerous cell proliferation in vitro [92]. NOX4 also advanced cancerous glycolytic reprogramming in renal carcinoma cells through the inhibition of PKM2 acetylation-mediated lysosomal-dependent degradation [106]. Importantly, in thyroid cells, NOX4-mediated glycolytic switch was also reflected in increased extracellular acidification rate, which is a read-out for lactate production [92]. One possible way to achieve hypoxia-independent activation of HIF-1α is by lactate [147]. Lactate is produced in the glycolysis, and several enzymes of this pathway are regulated by HIF-1α, providing a bidirectional regulatory configuration to adapt metabolic processes to hypoxic conditions [147]. Lactate produced and released by hypoxic cancer cells also serves as an intratumor metabolic fuel source preferentially used by oxygenated tumor cells (metabolic symbiosis) [130]. High tumor lactate is associated with increased risk of metastases and poor patient survival in head-and-neck cancers providing a possible link to NOX4 in these tumors [148]. 

Alongside HIF-1, another major coordinator of hypoxia-related adaptation is AMPK. AMPK is regarded as a key nutrient and energy sensor that synchronizes the adaptive response to energy stress [149]. AMPK is a heterotrimeric Ser/Thr kinase composed of one catalytic subunit (α) and two regulatory subunits (β and γ). The activation of AMPK relies on two intertwined mechanisms: the direct allosteric activation of the γ subunit by AMP (to a lesser extent ADP) and the reversible phosphorylation of the α subunit on its Thr172 residue. AMPK is phosphorylated by liver kinase B1 (LKB1) in response to energy depletion and by Ca^2+^/calmodulin-dependent kinase β (CaMKKβ) upon an increase in cytoplasmic Ca^+^ concentrations. Dephosphorylation (inactivation) of AMPK is elicited by protein phosphatases 2A and 2Cα (PP2A and PP2Cα). AMPK phosphorylation is a prerequisite for AMP-induced activation. However, once bound, AMP furthers AMPK phosphorylation by inducing conformational changes that protect AMPK against dephosphorylation [150]. ROS-mediated signals can increase AMPK activity both by a direct and an indirect way in a concentration-dependent manner. Physiological amounts of ROS might directly activate AMPK by a non-canonical pathway through oxidation or the glutathionylation of two cysteine residues (Cys299/Cys304) of AMPKα [151]. However, pathologically elevated ROS concentrations might lead to AMPK activation in an indirect fashion by inhibiting mitochondrial ATP synthesis with a consequent rise in AMP levels [152]. AMPK regulates a large variety of metabolic processes and controls mitochondrial health [149,153]. Concerning metabolism, activated AMPK enhances ATP-producing catabolic processes, including glucose uptake and glycolysis, and FA uptake and β-oxidation, and it suppresses ATP-consuming anabolic processes, such as gluconeogenesis and glycogen storage as well as FA, cholesterol and protein synthesis [132]. Through these actions, in non-cancerous cells, AMPK coordinates available nutritional resources to support cell endurance and advances stress resistance. In line with these functions, operational AMPK signaling is essential for cell survival under metabolic strain [30]. However, the role of AMPK in cancerous cells is more complex and context dependent [30]. Indeed, metabolic plasticity mediated by enhanced AMPK signaling might provide an initial advantage for tumor cells to confront their nutrient-poor environment. Conversely, however, the loss of AMPK signaling and thus a loss of cellular energy sensing might be beneficial for oncogenic proliferation by removing AMPK inhibitory effects on HIF-1-mediated glycolytic shift [154,155]. Diminished AMPK activity in high-glucose conditions was reported to enhance NOX4-dependent ROS production that contributed to human colon cancer cell growth and invasiveness [34]. The importance of uncovering signaling interactions between NOX4 and AMPK was also highlighted by data demonstrating that the pharmacological activation of AMPK suppresses mitochondrial oxidation and primes mitochondria apoptosis, leading to lessened tumor burden of acute myeloid lymphoma in mice [156].

Hypoxia induces neovascularization, the directed ingrowth of newly formed capillaries in the tumor mass to cater to the enhanced need of tumor cells for oxygen and nutrients. Neovascularization requires adequate hypoxia sensing, HIF-1α stabilization, and the expression of pro-angiogenic genes, e.g., VEGF and the glucose transporter 1 (GLUT1) [157]. Genetic NOX4 deficiency hampered these processes, resulting in slower growth of fibrosarcomas in a chemically induced tumor model in mice in vivo [158]. In addition, in vitro NOX4 enhanced retention of the VEGF receptor 2 (VEGFR-2) on the cell surface of endothelial cells, contributing to their targeted migration [159]. In vivo, NOX4 expression was detected in mouse brain neo-capillaries upon ischemia insult, suggesting that NOX4 plays a key role in hypoxia-induced capillary formation [160].

Redox-sensitive transcription factors are induced in response to elevations in cellular ROS levels and are key elements in diverse adaptive mechanisms that allow cancer cell survival, proliferation, and propagation [37,38,161]. Two main transcription factors whose signaling has been related to NOX4-mediated ROS in particular are NFκB and Nrf2 [35,36].

The members of the NFκB family of transcription factors enhance the transcription of a large number of genes that modulate cellular survival, proliferation, and apoptosis [162]. In its uninduced state, NFκB activity is repressed by association with its inhibitor IκB. Following the onset of oxidative stress, IκB undergoes phosphorylation, triggering its degradation and the liberation of NFκB. Once released, NFκB translocates to the nucleus and initiates the transcription of its target genes [163,164,165]. NFκB regulates several metabolic pathways to promote oncogenesis. In particular, NFκB upregulates hexokinase 2 expression and represses mitochondrial oxidative phosphorylation with a net outcome of promoting the Warburg effect [166,167]. Dysregulation within the NFκB signaling pathway is observed in diverse cancer types, and modulation of this pathway was suggested as a possible novel approach in cancer therapy [37,168,169]. In melanoma cells, NOX4-derived ROS production was described as a promoter of NFκB activation, sustaining cancer progression and metastasis [35].

Nrf2 is a member of a family of conserved proteins that are essential components of the cellular defense mechanism against oxidative stress [170,171]. Under unstressed conditions, Nrf2 is restrained in the cytosol by its associated inhibitor termed Kelch-like ECH-associated protein (KEAP1). When associated, KEAP1 facilitates the ubiquitination and subsequent proteasomal degradation of Nrf2 [172,173,174]. An increase in cellular ROS induces oxidation and the successive dissociation of KEAP1 from Nrf2, which then allows Nrf2 nuclear translocation [175,176]. Once in the nucleus, Nrf2 initiates the gene transcription of diverse genes of antioxidant defense [177,178,179]. Interestingly, the Nrf2–KEAP1 complex has also been detected in conjunction with the outer mitochondrial membrane, potentially allowing to combat increased mitochondrial ROS in a direct manner [180,181,182,183]. Nrf2-induced antioxidant gene transcription comprises genes of all four metabolic enzymes that generate NADPH and enzymatic components, maintaining functional glutathione and Trx antioxidant systems [184]. Indeed, Nrf2 upregulates expressions of G6PD, PGD, ME1, and IDH1, heightening cellular NADPH levels [185,186,187,188]. Concerning the glutathione system, Nrf2 controls key enzymes of glutathione biosynthesis (glutamate–cysteine ligase, (GCL), and glutathione S-transferases (GST)) and glutathione reduction (glutathione peroxidase (GPX2) and glutathione reductase (GR)) [189,190,191,192]. In addition, Nrf2 upregulates the expressions of TXN and thioredoxin reductase [193,194]. The mutation of *Nrf2* or *Keap1* can disrupt their interactions, leading to enhanced signaling by Nrf2 [195]. Nrf2 overactivation has been linked to diverse aspects of cancer cell auto-protection mechanisms and tumor development. Indeed, Nrf2 induces the expression of ROS-scavenging genes (e.g., catalase, GST, and Txn/Prx) and promotes GSH production and recycling [196,197,198,199]. Concerning NOX-mediated signaling, Nrf2 mediates redox adaptation in NOX4-overexpressed non-small cell lung (NSCL) cancer cells [36]. 

**Figure 6:**
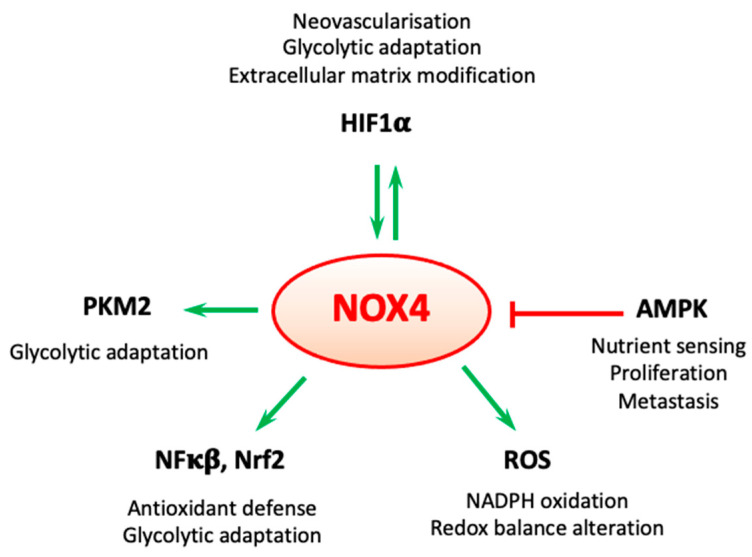
NOX4 in the center of carcinogenic metabolic rewiring. NOX4-mediated signals abet diverse signaling pathways to promote carcinogenesis. HIF1α promotes NOX4 expression, and conversely, NOX4-mediated ROS is essential for HIF1α-related glycolytic gene transcription and neovascularization through VEGF expression [142,158]. PKM2 requires NOX4 to achieve for glycolytic switch in certain cancer types [92]. NOX4 aides the activation of the transcription factors NFκB and Nrf2 [35,36]. NOX4-derived ROS directly modifies cellular redox balance and consumes the main antioxidant molecule NADPH. AMPK links nutrient sensing to NOX4. Indeed, AMPK inhibits the hyperglycemia-related upregulation of NOX4 expression and hampers the NOX4-related upregulation of p53 and apoptosis in kidney podocytes [31]. Green arrows: enhancing effect, red line: inhibitory effect.

A summary of the different signaling molecules modulating NOX4-related cancer cell proliferation, metabolic adaptation, and survival are depicted in Figure 6. 

## 4. NOX4 as a Therapeutic Target in Cancers

Unrestrained NOX4 activity has been described in diverse cancers, and NOX4 is now considered as a driving force in several cancer types [10]. In particular, upregulated NOX4 protein expression was identified by tissue microarray analysis in a series of malignancies including bladder, esophageal, head and neck, ovarian, and prostate carcinomas and malignant melanoma [20]. Upregulated NOX4 expression mediated oncogenic proliferation in renal cell carcinoma, melanoma, glioblastoma, and in ovarian and pancreatic cancer [200,201,202,203,204]. In addition, increased NOX4 expression was related to poor prognosis in colorectal cancer and was put forward as a compelling pharmacologic target in digestive system malignancies [21,22]. Most interestingly, enhanced NOX4 expression was reported in thyroid cancers and in thyroid cancer cell lines. siRNA-mediated knockdown of NOX-4 abolished the upregulation of several metabolic enzymes involved in cancer cell adaptation [25,27,92]. Currently, the precise mechanism of NOX4-related oncogenesis in diverse cancer types is incompletely understood and is the focus of intensive research efforts. Taking into consideration the ubiquitous expression pattern of NOX4, the link between NOX4 and oncogenesis is likely multifaceted and cell context specific. 

Cellular redox tone is a defining component in the regulation of cell fate deciding between proliferation, growth arrest, or death [205]. Cancer cells can endure supraphysiological levels of ROS, owing to their elevated antioxidant capacity. This feature accounts for their resistance to oxidative stress and thus contributes to their ability for uncontrolled proliferation in spite of unfavorable circumstances [206]. Therapeutic approaches targeting tumor redox comprise two opposite attitudes. While some of these propositions consider elevating cancer cell ROS levels in order to eventually overcome the limits of their antioxidant defense systems, others imply decreasing ROS levels by using antioxidants or ROS source-specific inhibitors [74,207]. However, these general ROS-targeted methods might have limited success due to the important role of redox metabolism of anti-tumor immune cells within or around the tumor mass [208]. The complexity of tumor cellular composition and their redox-related alterations raised the necessity of precision targeting of different ROS sources.

Inhibitors of NOX enzymes have been shown to slow tumor growth and promote cancer cell death [25,26,27,106,143,209,210]. The clinical relevance of NOX inhibitors has been further validated in 2019 when the World Health Organization (WHO) acknowledged them as a novel therapeutic class with the root name “naxib” (NADPH oxidase inhibitors). Among the NOX inhibitors, those that specifically restrain NOX4 activity gained particular interest in cancer research due to the multiple links between NOX4-derived ROS production and diverse aspects of tumor promotion, dissemination, therapy resistance, and that some of these effects were related to the modulation of the metabolic adaptive responses of tumor cells [21,22,27,29,36,211,212,213,214,215,216,217]. NOX inhibitors continuously evolve to improve their NOX isoform specificity with the aim to ameliorate therapeutic targeting [218,219,220]. Currently, the efficient utilization of NOX inhibitors as anticancer drugs is hampered by the lack of adequate information on several important issues. Firstly, tumors are complex identities constituted of cancer cells, capillaries, connective tissue, and immune cells, and NOX4-related redox pathways affect their functioning with different and often opposing pro- or anticancer outcomes. Secondly, NOX4 is a ubiquitously expressed NOX isoform that mediates several physiologically critical cellular processes; thus, inhibition of its activity might trigger perturbed cell function in healthy, extra-cancerous tissues. Thirdly, cellular redox molecules form interactive networks; thus, the inhibition of NOX4, a specific component of this system, might reverberate on the overall redox tone and lead to unfavorable outcomes. Lastly, NOX4-mediated ROS modulates diverse aspects of cancer development, other than metabolic adaptation, most notably the signaling of tyrosine kinase receptors and oncogenes that are often driving forces behind cancer cell proliferation [25,221,222,223]. This complexity is reflected in the controversial results obtained by several studies that investigated the role of NOX4 in oncogenesis. Indeed, data obtained from in vitro studies and in vivo murine models of cancerogenesis and metastasis implicated NOX4 both as a pro-and anti-oncogenic factor [105,202,224]. A part of these opposing results might be attributed to the cellular distribution of NOX4. Indeed, studies that evaluated the total cellular levels of NOX4 found that NOX4 was associated with favorable prognostics in hepatocellular cancers (HCC) [225]. By contrast, when the levels of nuclear NOX4 were separately analyzed in histological samples of human HCC, high levels of nuclear NOX4 staining were correlated with poor patient outcomes [226]. These observations instigate further studies to uncover the cell-specific factors that govern NOX4 activity and to identify the molecular targets of NOX4 in different cancer identities. From a clinical standpoint, a recent study provided encouraging data demonstrating that the NOX4 inhibitor GKT137831 (Setanaxib) was capable of overcoming immunotherapy resistant tumor growth by suppressing the exclusion of CD8^+^ T-cells from the tumor cells’ environment in in vivo rodent models [227]. 

In conclusion, both cancer metabolism and redox systems remain promising therapeutic targets [130]. NOX4 provides an intriguing link between these two systems, validating further studies to afford detailed comprehension of NOX4-related signaling pathways, their cell-specific redox interaction networks, and the support they provide in the metabolic adaptation of different tumors.
